# La interconexión de la investigación médica y la social

**DOI:** 10.7705/biomedica.7675

**Published:** 2024-08-29

**Authors:** Fernando Pío de la Hoz-Restrepo

**Affiliations:** * Departamento de Salud Pública, Facultad de Medicina, Universidad Nacional de Colombia, Bogotá, D.C., Colombia Universidad Nacional de Colombia Departamento de Salud Pública Facultad de Medicina Universidad Nacional de Colombia Bogotá, D.C. Colombia

Es de larga data el conocimiento de que es necesaria una fuerte relación entre la investigación médica y la investigación social, para explicar mejor las características de salud de los individuos y de las poblaciones. A mediados del siglo XIX, Rudolf Virchow, eminente figura de la investigación médica y fundador de la rama de la patología médica, sostenía que las causas de una epidemia de tifus entre los mineros de Silesia eran más sociales que médicas. En su informe sobre las causas de esa enfermedad en esa población, Virchow sostenía que las condiciones de pobreza extrema, suciedad y desnutrición en que vivían los mineros y sus familias, eran las principales causas de las enfermedades que padecían ^1^. Virchow, citado por Maciocco, sostenía que “la medicina es una ciencia social y la política no es más que medicina a gran escala” ^2^.

Además del trabajo de Virchow, se debe recordar que, desde el siglo XVIII y hasta la última mitad del siglo XIX, cuando la Revolución Industrial atiborró las principales ciudades de Europa occidental con cientos de miles de personas viviendo en condiciones precarias, varios trabajos de investigación sanitaria y económica demostraron la relación entre las cifras de mortalidad por diferentes causas y el vivir en condiciones de mayor o menor pobreza. En el siglo XVII, John Graunt -que no era médico sino una especie de contador- indicó que la mortalidad era mayor y más temprana en los habitantes de Londres que en quienes vivían en el campo, y que esa diferencia podría deberse a que las ciudades se habían ido transformando en lugares insalubres ^3^. William Farr, en la segunda mitad del siglo XIX, estableció los registros de mortalidad en Inglaterra y evidenció que ese evento variaba según los grupos humanos que desempeñaban diferentes profesiones, con lo cual comprobó la importancia de la ocupación en la salud humana; además, fue uno de los abanderados más importantes de la teoría miasmática, que sostenía que las malas condiciones sanitarias en que vivían los pobres eran la causa fundamental de las enfermedades, por el envenenamiento del suelo, el aire y el agua ^4^.

Según José Tapia, la separación entre las ciencias sociales - especialmente la economía-y la medicina para el estudio de las causas de las enfermedades es un fenómeno reciente ^5^. Entre los trabajos pioneros que desde el punto de vista de la economía intentan explicar las causas de la enfermedad, podemos citar los trabajos de Federico Engels. Este erudito alemán demostró que las condiciones de vida de los obreros en Inglaterra estaban asociadas con una mayor mortalidad a edades más tempranas, en comparación con la de las clases sociales más favorecidas. Asimismo, en diferentes partes de su obra, Carlos Marx resaltó la asociación entre el suicidio y las recesiones económicas -previamente descritas por Jacques Peuchet en Francia-y entre el crecimiento de la población y las malas condiciones de vida de los obreros.

Desde mediados del siglo XX, el mundo ha venido enfrentando el crecimiento de la carga de la enfermedad debida a afecciones cardiovasculares, cáncer de diferentes órganos y alteraciones de la salud mental. Todas estas enfermedades tienen causas biológicas y, también, sociales y económicas. Para explicar mejor el comportamiento de esos problemas, es necesario combinar diferentes enfoques para su estudio, incluyendo el fortalecimiento de la investigación sobre los factores determinantes sociales y económicos que influyen en ellas ^6^.

Existen diversas corrientes en el campo de la investigación científica que promueven una mejor aproximación para estudiar el papel que las condiciones sociales desempeñan en el desarrollo y el control de los problemas sanitarios mencionados. Desde la epidemiología y citando a Nancy Krieger ^7^, estos enfoques pueden agruparse en las siguientes categorías o teorías, como las llama esta autora:

1. *Teoría psicosocial*: sostiene que la interacción entre los humanos genera condiciones en el “ambiente social” que favorecen la aparición de diferentes enfermedades. La teoría propone una serie de factores psicosociales que considera relevantes para producir condiciones adversas en la sociedad, entre los cuales se cuentan las jerarquías sociales dominantes, la desorganización y los cambios sociales rápidos.
2. *Producción social de la enfermedad o economía política de la salud*: critica el enfoque epidemiológico clásico que pone el énfasis causal de las enfermedades crónicas y mentales en los hábitos de los individuos-“culpar a la víctima”-y propone, en cambio, que son las condiciones políticas y económicas las que producen las barreras estructurales que les impiden a las personas y a las comunidades vivir vidas saludables.
3. *Teoría ecosocial y perspectivas dinámicas multinivel*: contrario a las otras teorías cuyas descripciones esquemáticas son estacionarias, esta teoría propone marcos dinámicos de interacción entre factores que están situados en diferentes niveles jerárquicos explicativos e influyen en la ocurrencia de los problemas sanitarios. Los marcos dinámicos explicativos más conocidos son: el marco de la teoría ecosocial, el marco de la ecoepidemiología y el marco de la perspectiva de sistema socioecológico.

Todas estas teorías proponen enfoques multidisciplinarios para estudiar y explicar el comportamiento de los problemas sanitarios más relevantes, pero difieren radicalmente en las soluciones que proponen para prevenir o mitigar su impacto.

En mi percepción, la primera y la última proponen modificaciones a los sistemas económicos y sociales imperantes -capitalismo, sistemas de clases dominantes que discriminan por sexo, raza y otras características- sin atreverse a cambiarlos radicalmente. La segunda teoría, que se inspira fuertemente en el marxismo, propone, por el contrario, que se revisen y se cambien de arriba a abajo todos los esquemas de explotación económica y social que, según los propulsores de la teoría, generan modos de vida malsanos que oprimen a las personas y a las comunidades, y no permiten que accedan a mejores condiciones de vida lo que, a su vez, permitiría evitar muchos de los problemas sanitarios contemporáneos.

A mi modo de ver, los cambios graduales en los sistemas económicos actuales, que los lleven a ser más incluyentes y progresivos, son la mejor carta para intervenir en esas condiciones sociales y económicas que influyen desfavorablemente en la salud de las personas; sin embargo, no existe realmente mucha evidencia contemporánea sobre cómo llevar a cabo esos cambios pacíficamente, ni sobre la efectividad que puedan tener para disminuir la carga de la enfermedad.
